# Human Papillomaviruses and Malignant Neoplasms of the Female Upper Reproductive Tract: A Comprehensive Review of the Literature

**DOI:** 10.3390/cancers17121995

**Published:** 2025-06-15

**Authors:** Charalampos Karachalios, Ilias Liapis, Stamatios Petousis, Emmanouela-Aliki Almperi, Chrysoula Margioula-Siarkou, Georgia Margioula-Siarkou, Stefanos Flindris, Evangelos Karamitrousis, Konstantinos Dinas

**Affiliations:** 1 Gynecologic Oncology Unit, Second Department of Obstetrics and Gynecology, Aristotle University of Thessaloniki, 54124 Thessaloniki, Greece; petousisstamatios@gmail.com (S.P.); alemma300@gmail.com (E.-A.A.); margioulasiarkouc@gmail.com (C.M.-S.); gmargioulasiarkou@gmail.com (G.M.-S.); stefflindris@gmail.com (S.F.); konstantinosdinas@hotmail.com (K.D.); 2 Birmingham Women’s Hospital, Birmingham B15 2TG, West Midlands, UK; liapisilias7@hotmail.com; 3 International Oncology Center, Interbalkan Medical Center, 55535 Thessaloniki, Greece; vkaramitrousis@gmail.com

**Keywords:** human papillomavirus, endometrial cancer, uterine corpus cancer, uterine sarcoma, fallopian tube cancer, ovarian cancer, primary peritoneal cancer

## Abstract

Malignancies of the female upper reproductive tract, especially endometrial and ovarian cancers, generate a significant burden for women worldwide. The possible etiopathogenetic role of chronic human papillomavirus (HPV) infection in the carcinogenesis of the female upper genital tract is neither clearly established not completely understood. Therefore, we performed a literature review, using the PubMed and SCOPUS electronic databases, of the prevalence of HPV DNA in endometrial, primary fallopian tube, ovarian, and primary peritoneal cancers, as well as uterine sarcomas. The present investigation covered 35 studies from different countries on various continents. Overall, the prevalence of HPV was approximately 15% in all the above cancers. HPV DNA was isolated from 11%, 0%, 0%, and 14% of endometrial carcinomas, uterine sarcomas, primary fallopian tube cancers, and ovarian malignant neoplasms, respectively. No relevant studies on primary peritoneal cancers were retrieved. The predominant HPV strain from tumors of the upper female reproductive tract, regardless of the tumor site, was HPV-16, followed by HPV-18. The HPV DNA identified was exclusively from subtypes HPV-6, HPV-11, HPV-16, HPV-18, and HPV-33, which are responsible for the development of not only cervical cancer, but also condylomata acuminata. The findings of the present review indicate that HPV vaccination might prove to be a useful strategy in the prevention of HPV-related carcinomas of the upper genital tract in women.

## Simple Summary

Chronic human papillomavirus (HPV) infection has not been clearly established as the protagonist of upper female genital tract carcinogenesis. The principal aim of this comprehensive review is to determine the prevalence of HPV DNA isolated from malignant tumors of the uterus (excluding the cervix), oviducts, ovaries, and peritoneum. HPV DNA has been detected in endometrial and ovarian cancers. Consequently, HPV vaccination might prove to be a useful weapon in the armament against gynecological malignancies occurring in anatomical sites above the cervix uteri.

## 1. Introduction

The female upper reproductive tract consists of the cervix, uterus, fallopian tubes, and ovaries [1]. Malignant transformation in these structures could occur in each of the above sites, leading to the development of cancer, i.e., cancer of the cervix uteri [2], cancer of the corpus uteri [3], uterine sarcoma [4], primary fallopian tube cancer, ovarian cancer, or primary peritoneal cancer [5], respectively. The global burden of these diseases is significant. According to the Global Cancer Observatory (GLOBOCAN) of the International Agency for Research on Cancer (IARC) of the World Health Organization (WHO), a total of 662,301 new cervix uteri cancer cases were diagnosed in 2022 and a total of 348,874 deaths were attributed to this malignancy, whereas the global incidence and mortality of corpus uteri cancer in 2022 were 420,368 new cases and 97,723 deaths. Regarding ovarian cancer, 324,603 new cases were diagnosed in 2022 and 206,956 women died of the disease the same year [6].

The role of chronic human papillomavirus (HPV) infection in the development of cervical cancer was strongly proven and clearly established more than two decades ago. A seminal study by Walboomers et al., published in 1999 from the Netherlands, demonstrated that high-risk HPV DNA could be detected in nearly all (approximately 99.7%) cervical cancer cases worldwide, thus establishing HPV as a necessary cause of invasive cervical cancer [7]. Moreover, it was also later the same year that German virologist Harald zur Hausen published a comprehensive review of the evidence linking HPV infection to cervical carcinogenesis. zur Hausen’s pioneering work in the field laid the groundwork for future research, confirming the causal role of HPV in cervical cancer development [8]. Although HPV DNA has even been isolated from cancerous tissues in human body sites which are not directly or obviously linked to sexual intercourse, such as the lungs (non-small cell lung cancer, NSCLC) [9] and brain (glioblastoma multiforme, GBM) [10], the possible detrimental contribution of HPV infection to tumorigenesis in the female upper genital tract at an anatomical level higher than the cervix uteri still remains controversial, as the current published literature is divergent [11,12].

The aim of this comprehensive review is to address the prevalence of HPV DNA isolated from malignant neoplasms of the uterus, fallopian tubes, ovaries, and peritoneum and to attempt to correlate the presence of the HPV strains identified in these sites with the histopathology of the above malignancies, according to the data obtained from individual studies.

## 2. Materials and Methods

This is a comprehensive review of the literature that aims to summarize the current evidence regarding the prevalence of HPV DNA isolated from malignant tumors of the female upper reproductive tract, excluding the cervix uteri, i.e., epithelial and mesenchymal tumors of the corpus uteri, fallopian tube tumors, ovarian tumors, and primary peritoneal tumors [13]. The present review was conducted following the preferred reporting items for systematic reviews and meta-analysis (PRISMA) recommendations [14].

### 2.1. Data Sources and Search Strategy

A search of the literature was conducted on 22 April 2025 through the PubMed and SCOPUS electronic databases and designed using the “Systematic Review Accelerator” [15]. The literature search was performed for the period from 1 January 1985 to 22 April 2025 and searched for finally published, full-text English journal articles including human adult female individuals of >19 years of age. The search strategy was formed by combining appropriate MeSH terms and keywords with the help of the Boolean operators “OR” and “AND”. The reference lists of pertinent articles were further manually searched for potentially eligible results. The “Related Citations” tool in PubMed was also used whenever a suitable article was included. Table 1 presents the exact search string used for each database and each cancer site.

### 2.2. Eligibility Criteria

The inclusion criteria were (i) that the papers were prospective or retrospective randomized controlled trials (RCTs), cohort studies, case–control studies, case series, or case report studies of (ii) human female adult patients aged >19 years who had been histologically diagnosed with upper female reproductive tract cancer, irrespective of tumor stage, grade, and histologic type, that discussed (iii) HPV DNA extracted from the tumors and detected by polymerase chain reaction (PCR).

### 2.3. Exclusion Criteria

The exclusion criteria were (i) results that were books or book chapters, conference abstracts, theses, press articles, expert reviews, narrative reviews, systematic reviews, meta-analyses, or medical hypotheses; (ii) animal studies, in vitro studies, abstract- or protocol-only publications or video reports; (iii) non-English articles and published abstracts without full-text manuscripts and results in which (iv) concomitant disease site(s) with precancerous or cancerous lesions were present; (iv) the HPV genome was extracted from samples other than malignant tissues from the upper female genital tract, such as blood; (v) patients and/or non-patients with prior neoplasia, surgery for malignancy, radiation or cytotoxic therapy were included; (vi) other oncogenic viruses were also present; and (vii) HPV was detected by methods other than PCR.

### 2.4. Study Screening and Selection Process

Two authors, CK and IL, independently selected the articles which fulfilled the inclusion criteria. Once the initial title and abstract screening was completed, the full texts of the studies included from that stage were reviewed by the aforementioned investigators to determine whether they should be included. Discrepancies were resolved by consulting a third author, SP. The aforementioned review stage was completed with the assistance of the automation tool “Systematic Review Accelerator” [15].

### 2.5. Data Extraction

**Figure 1:**
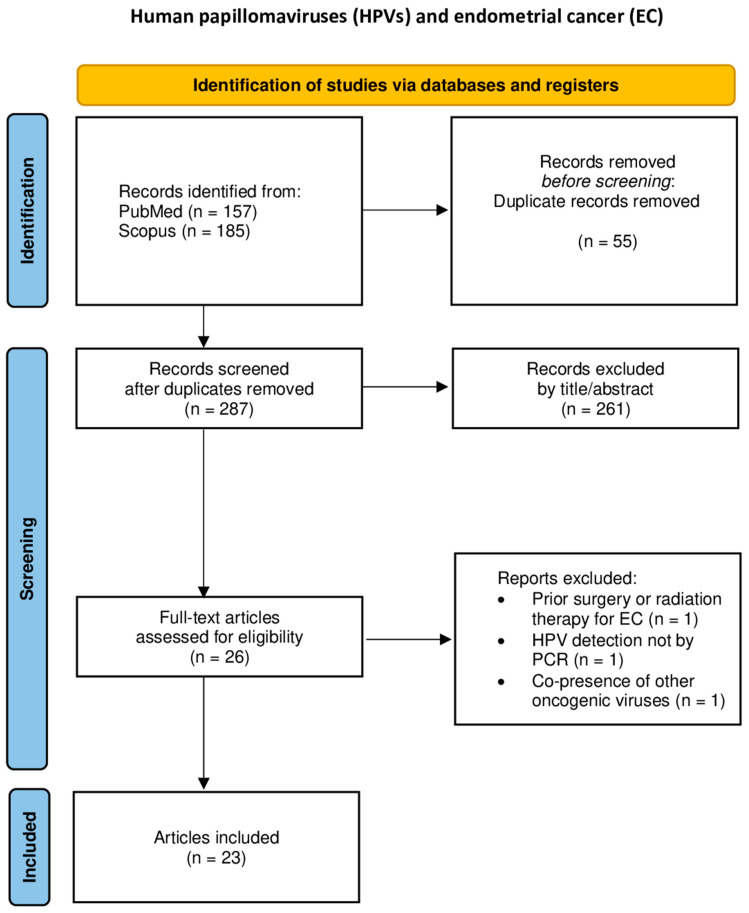
Flowchart of study selection for endometrial cancer.

**Figure 2:**
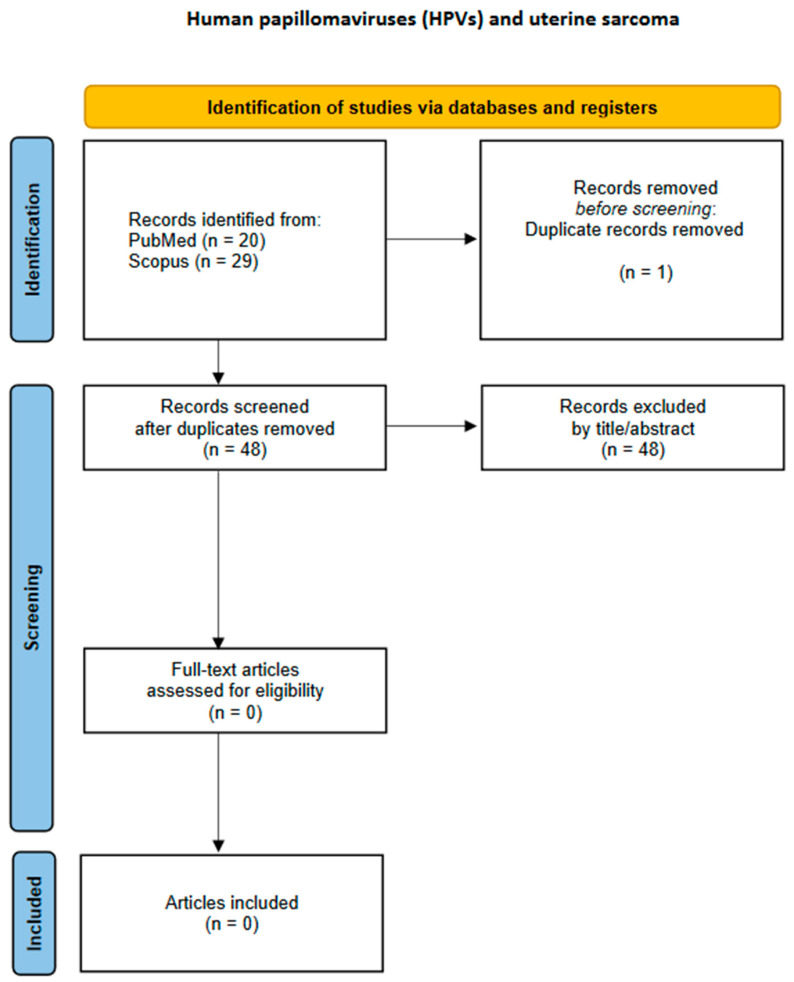
Flowchart of study selection for uterine sarcoma.

**Figure 3:**
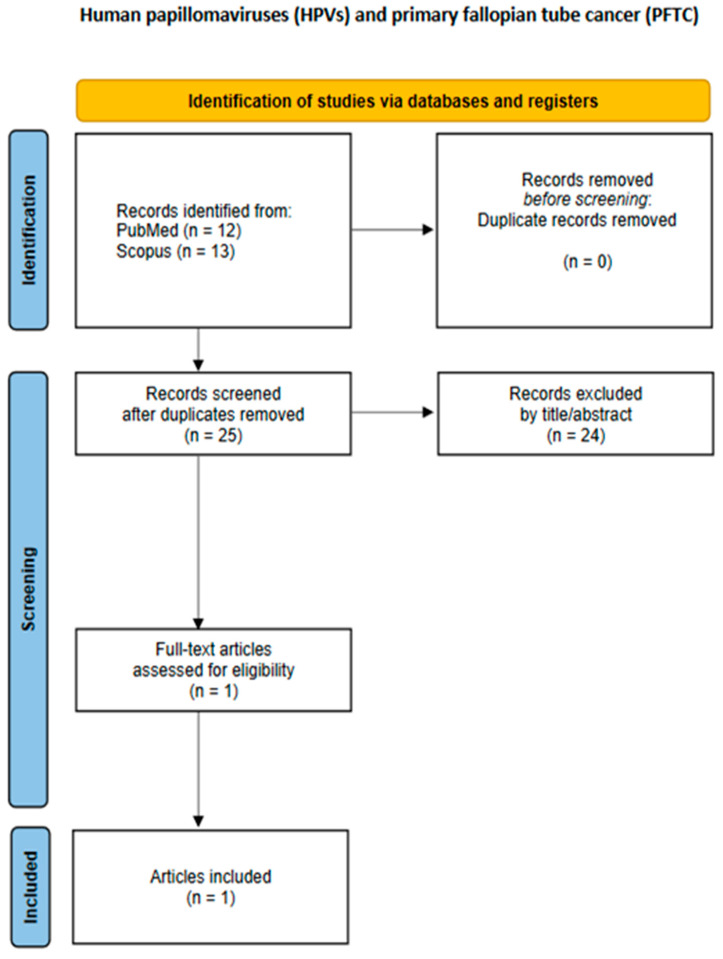
Flowchart of study selection for primary fallopian tube cancer.

**Figure 4:**
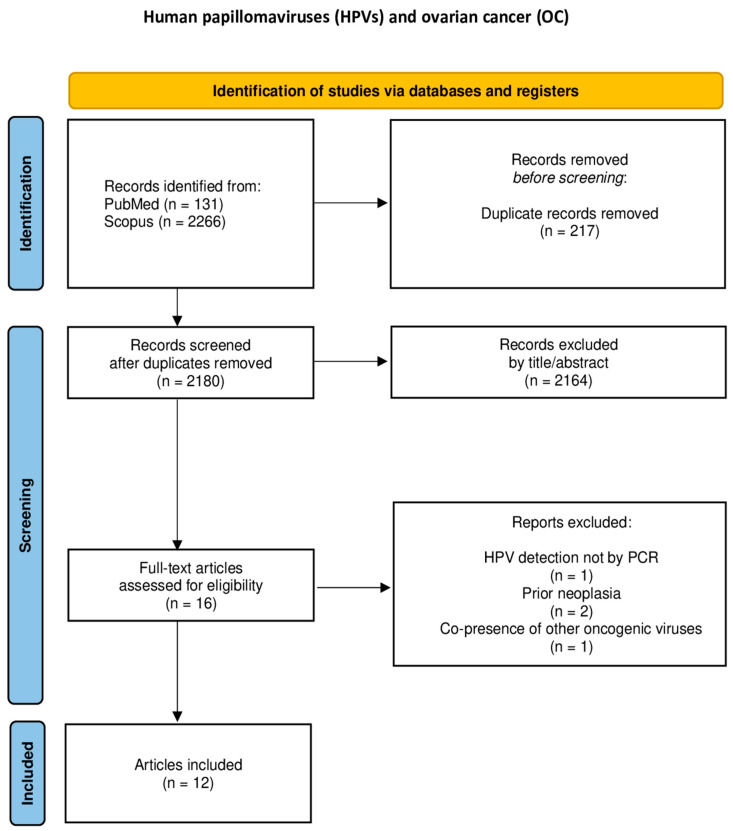
Flowchart of study selection for ovarian cancer.

**Figure 5:**
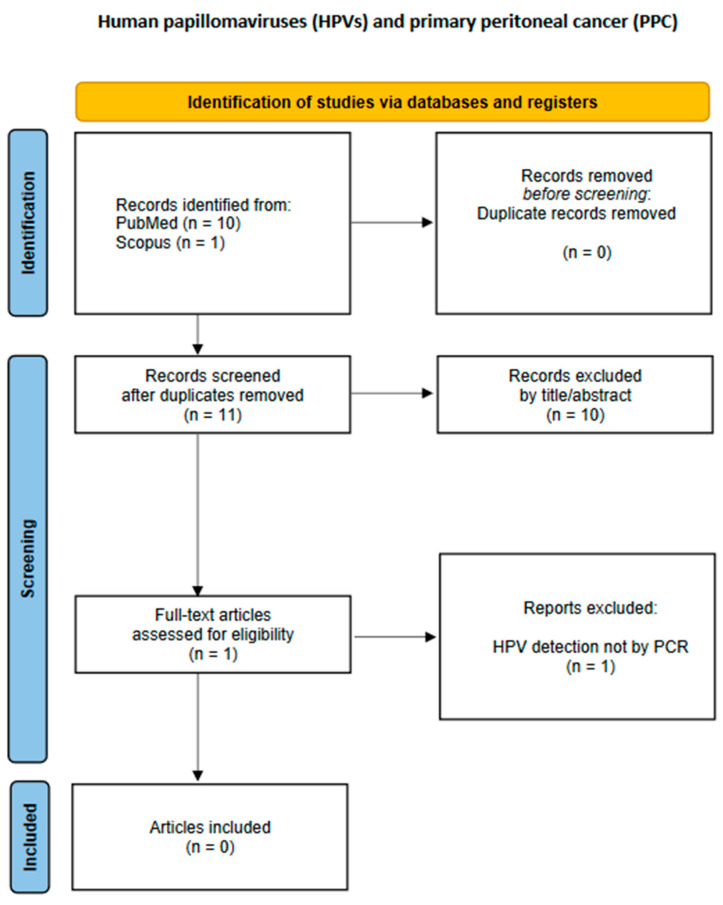
Flowchart of study selection for primary peritoneal cancer.

The study characteristics and data outcomes from each study were recorded in a data extraction form. Data extraction was independently conducted by two researchers, CK and EAA. A third and fourth researcher, CMS and GMS, were consulted to resolve disagreements through open discussion. The following data regarding study characteristics and outcomes were extracted from every included study: first author, publication year, type of study, malignant neoplasm(s) histopathologic type(s), HPV DNA positivity, and the HPV subtypes isolated from the tumors by PCR. The flowcharts of the study selection process for each malignancy site are presented in Figure 1, Figure 2, Figure 3, Figure 4 and Figure 5.

## 3. Results

An overview of all selected studies is presented in Table 2. A total of 35 articles, published from 1992 up to 2024, meet the eligibility criteria for the present comprehensive review. These consist of 8 case–control studies [16,17,18,19,20,21,22,23], 25 case series [11,12,24,25,26,27,28,29,30,31,32,33,34,35,36,37,38,39,40,41,42,43,44,45,46], and 2 case reports [47,48]. HPV DNA was isolated from 222 specimens out of 1,666 malignant neoplastic tissues (222/1,666; 13%). All 222 specimens originated exclusively from 101 and 189 of the endometrial and ovarian carcinomas tested, as no HPV DNA has been isolated from uterine sarcomas, primary fallopian tube carcinomas, or primary peritoneal carcinomas. Although various commercially available kits capable of identifying DNA from several different HPV strains were deployed for the isolation of viral DNA from the above tumors, HPV DNA was found to come from only subtypes HPV-6, HPV-11, HPV-16, HPV-18, and HPV-33. In general, the predominant HPV strain from all tumors of the upper female reproductive tract, regardless of the tumor site, was HPV-16 (145/222; 65%) followed by HPV-18 (48/222; 22%).

### 3.1. Endometrial Cancer (EC)

The presence of HPV DNA in EC was examined in 24 studies, i.e., 5 case–control studies [16,17,18,19,22], 17 case series [24,25,26,27,28,29,30,31,32,33,34,35,36,37,38,40,46], and 2 case reports [47,49], which included a total of 668 patients with EC. Endometrioid endometrial carcinomas were the most frequent histological type recorded. Viral DNA was detected in 15 of 24 studies (62%) and in 74 patients (74/668; 11%). HPV DNA positivity ranged from 0 to 100%. DNA from HPV-16 was isolated from 29 patients, making this the most common HPV subtype (29/74; 39%). The rest of the HPV strains detected were HPV-6, HPV-18, HPV-33, HPV-11, and HPV-31, which were found in 20, 12, 8, 3, and 2 EC specimens, respectively.

### 3.2. Uterine Sarcomas

Only one leiomyosarcoma was identified from a case series published in 1994 by Koffa et al., from which no HPV DNA was isolated [40].

### 3.3. Primary Fallopian Tube Cancer (PFTC)

In a case series of 7 PFTCs published in 1996 by Runnebaum et al., no HPV DNA was identified in these relatively rare malignant neoplasms [39].

### 3.4. Ovarian Cancer (OC)

The presence of HPV DNA in OC was examined in 16 studies, i.e., 4 case–control studies [20,21,22,23] and 12 case series [11,12,24,32,33,36,40,41,42,43,44,45], which included 996 patients with OC in total. HPV DNA was found in 13 of 16 studies (81%) and 148 patients (148/996; 15%). Ovarian serous adenocarcinomas were the most frequent histological type to display both HPV DNA-positive and -negative ovarian malignant neoplasms, accounting for more than half of all cases (542/996; 54%). HPV DNA positivity ranged from 0 to 62% [45]. DNA from HPV-16 was isolated from 116 patients, making this the most common HPV subtype (116/148; 78%). The rest of the HPV strains detected were HPV-18 and HPV-33, which were detected in 26 and 1 OC specimens, respectively.

### 3.5. Primary Peritoneal Cancer (PPC)

A study by Gatalica et al. published in 2008 described a case of low-grade peritoneal mucinous carcinomatosis containing high-risk HPV sequences (HPV-16, -18, -31, -33, -35, -39, -51, -52, -56, -58, and -66) [50]. However, it did not meet the eligibility criteria and thus had to be excluded, because the presence of HPV was not confirmed by PCR but by in situ hybridization, without exact HPV genotyping. Therefore, our search retrieved no relevant results.

## 4. Discussion

### 4.1. General Considerations

Evidence from the literature of the last forty years shows a very wide heterogeneity in the prevalence of HPV DNA isolated from malignant tissues of the upper female genital tract, which ranges from 0 [11] to 100% [38]. Of note, there are neither narrative nor systematic reviews focused exclusively on examining the potential role of HPVs in the development of uterine sarcomas, primary fallopian tube carcinomas, or primary peritoneal cancers, most probably due to the relative rarity of these malignant neoplasms in comparison to endometrial and ovarian cancer. The most frequently identified HPV subtype for all HPV DNA-positive malignancies studied was HPV-16, regardless of the geographical region from which the samples were collected, which is in agreement with several meta-analyses published by different researchers in different years [51,52,53,54]. Geographical differences in the prevalence of HPV DNA might be attributed to geographical and biological interactions between HPV subtypes and host immunogenetic factors, such as HLA (human leukocyte antigen), GST (glutathione-S-transferase), FAS (fatty acid synthase) gene promoter-670, MDM2 (Mouse double minute 2 homolog), and p53 codon 72 polymorphisms [55]. Moreover, the prevalence of HPV DNA can vary according to the condition of the tissue when studied (for example, frozen tissues versus formalin-fixed paraffin-embedded tissues, FFPE) and the detection method used (for example, PCR versus in situ hybridization; PCR is considered to be the most sensitive method for detecting HPV [51]) in each included study. In a meta-analysis from Denmark by Svahn et al., published in 2014, the prevalence of HPV was slightly higher in frozen tissues than FFPE tissues, but this difference was not statistically significant [51]. Paraffin-embedded tissues amplify DNA products less efficiently than fresh tissues due to a loss of DNA integrity, especially when fixation protocols including non-buffered formalin are implemented [51,54]. A long fixation time in formalin could be responsible for the cross-linking of nucleic acids and proteins, as well as random breaks in nucleotide sequences. Additionally, carryover contamination can occur if FFPE blocks are not carefully processed, thus leading to false positive results [54].

It is also worth mentioning that only three of the studies included in Table 2 mention the copy number of HPV-16 or HPV-18 DNA in some of their positive specimens, all of which originated from either endometrial or ovarian carcinomas. According to Yang et al. [36], “the median copy numbers of HPV 16 DNA in endometrial and ovarian cancers were 3,500 and 7,590 copies/μg DNA, respectively. These amounts were also significantly (*p* < 0.05) lower than HPV 16 DNA in cervical cancer (492,800 copies/μg DNA)”. Moreover, in a case series by Jiang et al. [26], “the tumors in the endometrium and the endocervix had similar results by HPV DNA by in situ hybridization, which has a detection threshold of 10 copies per cell”. Furthermore, in a recent study by Jarych et al. [45], “the median HPV16 DNA concentrations in cancerous ovarian samples were significantly higher (median 62.16 copies per 10^5^ cells, range 5.48–554.68 copies per 10^5^ cells) than those for HPV18 DNA concentrations (median 11.27 copies per 10^5^ cells, range 2.77–477.91 copies per 10^5^ We cells) (*p* = 0.0051, Mann–Whitney U test)”.

### 4.2. HPV and Endometrial Cancer

In the present review, endometrial cancer’s prevalence, regardless of type, was approximately 11%, ranging from 0% to 100%. Endometrioid EC was the most common histological type studied and HPV-16 was the most frequent HPV subtype found. According to a systematic review and meta-analysis from Denmark published by Olesen et al. in 2014, which included 1026 cases of EC from 29 studies, the prevalence of HPV DNA varied from 0% to 61% and the pooled prevalence of HPV DNA was 10%. The majority (97%, n = 761/1026) of endometrial carcinomas were Type 1 (endometrioid, endometrioid with squamous differentiation, villoglandular, secretory, ciliated cell, and mucinous) [52].

An important aspect of HPV-related endometrial carcinogenesis involves the route of HPV’s transmission from the lower female genital tract to the upper female reproductive tract. The former includes the vagina and the ectocervix, is covered with multiple layers of stratified squamous epithelia, and is often exposed to pathogens. The latter, which includes the uterus, endometrium, and endocervix, consists of a single layer of columnar epithelium that maintains a relatively sterile environment, with intermittent microorganisms ascending from the lower female genital tract. The endocervix functions as an interface between the relatively sterile upper female reproductive tract and the non-sterile lower female genital tract. It is hypothesized that HPVs could ascend to the endometrium from the vagina and the ectocervix through the endocervix and infect the squamous epithelium in the endometrium. It is in a similar fashion that HPV infects the basal cells of the squamous epithelium in the cervix [52].

### 4.3. HPV and Ovarian Cancer

The first report on HPV infection in OC was published in 1987 by Kaufmann et al., whose research team had used Southern blot hybridization [55], and the first report on HPV-related primary ovarian squamous cell carcinoma was published by Mai et al. in 1996 [56]. Since then, several reports have examined the prevalence of HPV DNA in OC. In a meta-analysis by Svahn et al., the prevalence of HPV in ovarian carcinomas ranged from 2% to 67% and its pooled prevalence was approximately 16%. HPV-16, which was the most frequently isolated HPV subtype, was found in serous, mucinous, and endometrioid ovarian malignant tissues [51]. Moreover, in a meta-analysis from Brazil published by Rosa et al. in 2013, 17.5% of OC cases reported an HPV infection. HPV-16 was also the most common strain, followed by HPV-18. Only subtypes 6, 16, 18, and 33 were identified [53]. Furthermore, a more recent meta-analysis by Ibragimova et al., which included 14 studies and a total of 1163 OC samples, revealed a low risk of OC development with HPV infection. The prevalence of HPV in OC averaged 22%, ranging from 0% to 90% [54]. Similar findings were obtained in a meta-analysis from Berlin, Germany, by Cherif et al., which was published in the same year and included 2280 cases of OC, in which the prevalence of detection ranged from 0% to 81% and the overall pooled prevalence of HPV was approximately 16% [55].

A variety of possible transmission pathways have been hypothesized to lead to the detection of HPV DNA in ovarian malignant tissues, such as (i) an ascending infection from the lower genital tract, as the endometrium and oviducts are an anatomical continuation of the endocervix [53]; (ii) semen containing HPV DNA and spermatozoa in contaminated semen absorbing HPV DNA and transmitting it to ovarian tissues; or (iii) lymphocytes transporting HPV to the ovaries. In each of the cases above, after the virus reaches the ovaries, the disruption of the ovarian epithelium during ovulation could facilitate an ovarian HPV infection [51]. HPV’s prevalence is significantly higher in squamous cell cervical tissues than the glandular tissues of the ovaries, thus indicating the possibly greater affinity of HPV with the former anatomical site [51]. Moreover, the cervical transformation zone (TZ) might be vulnerable to HPV infection because the TZ consists of cuboidal epithelial cells, reserve cells, or potentially embryonal stem cells, which are possible targets of HPV, whereas similar target cells have not been described in the ovaries, which potentially explains the overall lower prevalence of HPV DNA in OC compared to cervical cancer, as the ovaries are “protected” from HPV DNA’s integration into the ovarian cells’ genome [55].

## 5. Limitations

The present comprehensive review used a systematic method to retrieve as much evidence as possible that has been published in the last four decades on this issue. However, it has several limitations. First, it does not include older studies published prior to 1992. Second, only two electronic databases (PubMed and SCOPUS) were searched. Third, three of the included studies [23,40,44] do not clearly state which HPV subtype was isolated from the malignant tumors that were analyzed. Fourth, only 7 out of the 35 included studies were case–control studies. Therefore, we were not able to distinguish possible differences between malignant neoplasms and healthy tissues serving as controls. Fifth, not all studies reported which HPV strains were detected in the malignant tumors by their histological type. Sixth, the samples from some of the included studies were obtained from fresh-frozen paraffin-embedded tissue blocks, while the samples from other included studies were collected from fresh tissues. Seventh, possible contamination, from the cervix, of the examined malignant tissues could not be totally excluded in every included study, especially in cases where no HPV detection that was isolated from the cervix was carried out. Eighth, it was the possible presence of HPV DNA and not of HPV mRNA that was assessed in the included studies. HPV mRNA marks a transcriptionally active HPV infection [57], while HPV DNA shows the mere presence of the virus. Ninth, no studies of primary peritoneal cancer have been included in the present review. Last, but not least, there was no information on the HPV vaccination status of the patients and/or controls, especially in newer studies included in our review.

## 6. Implications for Practice and Future Research

HPV DNA isolation from the upper female genital tract varies widely among the published studies. Nevertheless, the presence of both high-risk and low-risk HPV subtypes in anatomical sites above the cervix implicates their potential role in the development of cancer at these sites. However, a more sensitive detection method, based on mRNA HPV oncogene expression instead of HPV DNA isolation, could compare the viral oncogene expression in malignant tissue samples with that in adjacent healthy tissues, thus further clarifying the potential role of HPVs in tumorigenesis in the upper female reproductive tract. Moreover, there is no strong evidence of whether HPV vaccines could lower the incidence of these malignant neoplasms. Not only prospective multicenter studies on the incidence of HPV DNA-positive malignant tumors in HPV-vaccinated versus HPV-unvaccinated women, but also large-sample retrospective studies on the prevalence of HPV vaccination in HPV DNA-positive malignancies of the upper female reproductive tract, could shed further light on the potentially protective role of HPV vaccines against carcinogenesis in the corpus uteri, adnexa, and peritoneum.

## 7. Conclusions

The present comprehensive review demonstrated that HPV DNA can be found in both endometrial carcinomas and ovarian cancer cases. Highly oncogenic HPV-16 and HPV-18 were the most common HPV strains identified in the majority of the studies. Therefore, the necessity of HPV vaccination for women and men remains imperative in ensuring that a significant reduction in the incidence and burden of HPV-related malignancies of the upper female genital tract is achieved worldwide.

## Figures and Tables

**Table 1:** Electronic database search string for each cancer site.

Malignancy Site	PubMed	SCOPUS
Endometrial cancer	((“human papillomavirus viruses”[MeSH Terms] OR “human papillomavirus”[Title/Abstract] OR “HPV”[Title/Abstract]) AND (“endometrial neoplasms”[MeSH Terms] OR “endometrial neoplasms”[MeSH Terms] OR (“endometrial”[All Fields] AND “neoplasms”[MeSH Terms]) OR “endometrial cancer*”[Title/Abstract] OR “endometrial carcinoma*”[Title/Abstract] OR (“endometrial”[All Fields] AND “malignant neoplasm*”[Title/Abstract]) OR “endometrial ca”[Title/Abstract])) AND ((excludepreprints[Filter]) AND (fft[Filter]) AND (humans[Filter]) AND (female[Filter]) AND (1985/1/1:2025/1/1[pdat]) AND (english[Filter]) AND (alladult[Filter]))	(INDEXTERMS(“human papillomavirus viruses”) OR TITLE-ABS(“human papillomavirus”) OR TITLE-ABS(HPV)) AND (INDEXTERMS(“endometrial neoplasms”) OR INDEXTERMS(“endometrial neoplasms”) OR (ALL(endometrial) AND INDEXTERMS(neoplasms)) OR TITLE-ABS(“endometrial cancer*”) OR TITLE-ABS(“endometrial carcinoma*”) OR (ALL(endometrial) AND TITLE-ABS(“malignant neoplasm*”)) OR TITLE-ABS(“endometrial ca”)) AND PUBYEAR > 1985 AND PUBYEAR < 2026 AND (LIMIT-TO (DOCTYPE,”ar”)) AND (LIMIT-TO (PUBSTAGE,”final”)) AND (LIMIT-TO (SRCTYPE,”j”)) AND (LIMIT-TO (LANGUAGE,”English”))
Uterine sarcoma	((“human papillomavirus viruses”[MeSH Terms] OR “human papillomavirus”[Title/Abstract] OR “HPV”[Title/Abstract]) AND (((“uterin”[All Fields] OR “uterines”[All Fields] OR “uterus”[MeSH Terms] OR “uterus”[All Fields] OR “uterine”[All Fields]) AND “sarcoma”[MeSH Terms]) OR “uterine sarcoma*”[Title/Abstract])) AND ((excludepreprints[Filter]) AND (fft[Filter]) AND (humans[Filter]) AND (female[Filter]) AND (1985/1/1:2025/1/1[pdat]) AND (english[Filter]) AND (alladult[Filter]))	(INDEXTERMS(“human papillomavirus viruses”) OR TITLE-ABS(“human papillomavirus”) OR TITLE-ABS(HPV)) AND (((ALL(uterin) OR ALL(uterines) OR INDEXTERMS(uterus) OR ALL(uterus) OR ALL(uterine)) AND INDEXTERMS(sarcoma)) OR TITLE-ABS(“uterine sarcoma*”)) AND PUBYEAR > 1984 AND PUBYEAR < 2026 AND (LIMIT-TO (DOCTYPE,”ar”)) AND (LIMIT-TO (PUBSTAGE,”final”)) AND (LIMIT-TO (SRCTYPE,”j”)) AND (LIMIT-TO (LANGUAGE,”English”))
Fallopian tube cancer	((“human papillomavirus viruses”[MeSH Terms] OR “human papillomavirus”[Title/Abstract] OR “HPV”[Title/Abstract]) AND (“ovarian neoplasms”[MeSH Terms] OR “ovarian neoplasms”[MeSH Terms] OR ((“ovarian”[All Fields] OR “ovarians”[All Fields]) AND “neoplasms”[MeSH Terms]) OR “ovarian cancer*”[Title/Abstract] OR “ovarian carcinoma*”[Title/Abstract] OR “ovarian malignant neoplasm*”[Title/Abstract] OR “ovarian ca”[Title/Abstract])) AND ((excludepreprints[Filter]) AND (fft[Filter]) AND (humans[Filter]) AND (female[Filter]) AND (1985/1/1:2025/1/1[pdat]) AND (english[Filter]) AND (alladult[Filter]))	(INDEXTERMS(“human papillomavirus viruses”) OR TITLE-ABS(“human papillomavirus”) OR TITLE-ABS(HPV)) AND (INDEXTERMS(“fallopian tube neoplasms”) OR TITLE-ABS(“fallopian tube cancer*”) OR TITLE-ABS(“fallopian tube carcinoma*”) OR ((INDEXTERMS(“fallopian tubes”) OR (ALL(fallopian) AND ALL(tubes)) OR ALL(“fallopian tubes”) OR (ALL(fallopian) AND ALL(tube)) OR ALL(“fallopian tube”)) AND TITLE-ABS(“malignant neoplasm*”))) AND PUBYEAR > 1995 AND PUBYEAR < 2022 AND (LIMIT-TO (DOCTYPE,”ar”)) AND (LIMIT-TO (PUBSTAGE,”final”)) AND (LIMIT-TO (SRCTYPE,”j”)) AND (LIMIT-TO (LANGUAGE,”English”))
Ovarian cancer	((“human papillomavirus viruses”[MeSH Terms] OR “human papillomavirus”[Title/Abstract] OR “HPV”[Title/Abstract]) AND (“ovarian neoplasms”[MeSH Terms] OR “ovarian neoplasms”[MeSH Terms] OR ((“ovarian”[All Fields] OR “ovarians”[All Fields]) AND “neoplasms”[MeSH Terms]) OR “ovarian cancer*”[Title/Abstract] OR “ovarian carcinoma*”[Title/Abstract] OR “ovarian malignant neoplasm*”[Title/Abstract] OR “ovarian ca”[Title/Abstract])) AND ((excludepreprints[Filter]) AND (fft[Filter]) AND (humans[Filter]) AND (female[Filter]) AND (1985/1/1:2025/1/1[pdat]) AND (english[Filter]) AND (alladult[Filter]))	(INDEXTERMS(“human papillomavirus viruses”) OR TITLE-ABS(“human papillomavirus”) OR TITLE-ABS(HPV)) AND (INDEXTERMS(“ovarian neoplasms”) OR INDEXTERMS(“ovarian neoplasms”) OR ((ALL(ovarian) OR ALL(ovarians)) AND INDEXTERMS(neoplasms)) OR TITLE-ABS(“ovarian cancer*”) OR TITLE-ABS(“ovarian carcinoma*”) OR TITLE-ABS(“ovarian malignant neoplasm*”) OR TITLE-ABS(“ovarian ca”)) AND PUBYEAR > 1986 AND PUBYEAR < 2026 AND (LIMIT-TO (DOCTYPE,”ar”)) AND (LIMIT-TO (PUBSTAGE,”final”)) AND (LIMIT-TO (SRCTYPE,”j”)) AND (LIMIT-TO (LANGUAGE,”English”))
Primary peritoneal cancer	((“human papillomavirus viruses”[MeSH Terms] OR “human papillomavirus”[Title/Abstract] OR “HPV”[Title/Abstract]) AND (((“primaries”[All Fields] OR “primary”[All Fields]) AND (“peritoneally”[All Fields] OR “peritoneum”[MeSH Terms] OR “peritoneum”[All Fields] OR “peritoneal”[All Fields] OR “peritonism”[All Fields] OR “peritonitis”[MeSH Terms] OR “peritonitis”[All Fields]) AND “neoplasms”[MeSH Terms]) OR ((“primaries”[All Fields] OR “primary”[All Fields]) AND (“peritoneally”[All Fields] OR “peritoneum”[MeSH Terms] OR “peritoneum”[All Fields] OR “peritoneal”[All Fields] OR “peritonism”[All Fields] OR “peritonitis”[MeSH Terms] OR “peritonitis”[All Fields]) AND “carcinoma”[MeSH Terms]) OR “primary peritoneal cancer*”[Title/Abstract] OR “primary peritoneal carcinoma*”[Title/Abstract] OR ((“primaries”[All Fields] OR “primary”[All Fields]) AND (“peritoneally”[All Fields] OR “peritoneum”[MeSH Terms] OR “peritoneum”[All Fields] OR “peritoneal”[All Fields] OR “peritonism”[All Fields] OR “peritonitis”[MeSH Terms] OR “peritonitis”[All Fields]) AND “malignant neoplasm*”[Title/Abstract]))) AND ((excludepreprints[Filter]) AND (fft[Filter]) AND (humans[Filter]) AND (female[Filter]) AND (1985/1/1:2025/1/1[pdat]) AND (english[Filter]) AND (alladult[Filter]))	(INDEXTERMS(“human papillomavirus viruses”) OR TITLE-ABS(“human papillomavirus”) OR TITLE-ABS(HPV)) AND (((ALL(primaries) OR ALL(primary)) AND (ALL(peritoneally) OR INDEXTERMS(peritoneum) OR ALL(peritoneum) OR ALL(peritoneal) OR ALL(peritonism) OR INDEXTERMS(peritonitis) OR ALL(peritonitis)) AND INDEXTERMS(neoplasms)) OR ((ALL(primaries) OR ALL(primary)) AND (ALL(peritoneally) OR INDEXTERMS(peritoneum) OR ALL(peritoneum) OR ALL(peritoneal) OR ALL(peritonism) OR INDEXTERMS(peritonitis) OR ALL(peritonitis)) AND INDEXTERMS(carcinoma)) OR TITLE-ABS(“primary peritoneal cancer*”) OR TITLE-ABS(“primary peritoneal carcinoma*”) OR ((ALL(primaries) OR ALL(primary)) AND (ALL(peritoneally) OR INDEXTERMS(peritoneum) OR ALL(peritoneum) OR ALL(peritoneal) OR ALL(peritonism) OR INDEXTERMS(peritonitis) OR ALL(peritonitis)) AND TITLE-ABS(“malignant neoplasm*”))) AND PUBYEAR > 1986 AND PUBYEAR < 2026 AND (LIMIT-TO (DOCTYPE,”ar”)) AND (LIMIT-TO (PUBSTAGE,”final”)) AND (LIMIT-TO (SRCTYPE,”j”)) AND (LIMIT-TO (LANGUAGE,”English”))

**Table 2:** Studies on the presence of HPV DNA in malignancies of the upper female reproductive tract.

First Author, Year, Reference	Study Type	Malignant Neoplasm Histopathologic Type	HPV Positivity
Anwar et al. 1996 [24]	Case series	15 endometrial carcinomas	• 2/15 (13%) HPV-18
			• 5/15 (33%) HPV-33
		3 ovarian carcinomas	None
O’Leary et al. 1998 [25]	Case series	20 endometrial adenocarcinomas	2/20 (10%) HPV-11
		41 endometrial adenocarcinomas with squamous metaplasia	• 19/41 (46%) HPV-6
			• 2/41 (5%) HPV-16
			• 1/41 (2%) HPV-18
			• 2/41 (5%) HPV-33
		2 adenosquamous endometrial carcinomas	1/2 (50%) HPV-6 and HPV-33
			(same patient)
Jiang et al. 2010 [26]	Case series	4 endometrioid endometrial adenocarcinomas	• 1/4 (25%) HPV-11
			• 1/4 (25%) HPV-18
Abu-Lubad et al. 2020 [16]	Case–control study	36 endometrial carcinomas	3/20 (15%) HPV-18
Bures et al. 2013 [27]	Case series	5 primary squamous cell carcinomas of the endometrium	None
		8 endometrioid endometrial carcinomas	
Fedrizzi et al. 2009 [17]	Case–control study	50 endometrial carcinomas	• 3/50 (6%) HPV-16
			• 1/50 (2%) HPV-18 and HPV-31 (same patient)
Fujita et al. 1995 [28]	Case series	85 endometrial adenocarcinomas	8/85 (9%) HPV-16
Giordano et al. 2006 [30]	Case series	2 mucinous microglandular endometrial adenocarcinomas	None
Giordano et al. 2005 [47]	Case report	1 primary squamous cell endometrial carcinoma	None
Hachisuga et al. 1996 [29]	Case series	30 endometrial carcinomas	None
Hording et al. 1997 [31]	Case series	23 endometrial carcinomas	None
Ip et al. 2002 [32]	Case series	55 endometrial adenocarcinomas	5/55 (9%) HPV-16
		60 primary epithelial ovarian carcinomas	6/60 (10%) HPV-16/18
		4 mucinous borderline ovarian tumors	• 3/60 (5%) HPV-16
			• 1/60 (2%) HPV-18
		1 clear-cell ovarian adenocarcinoma	1/60 (2%) HPV-16
		1 mucinous ovarian adenocarcinoma	1/60 (2%) HPV-16
Karadayi et al. 2013 [18]	Case–control study	30 endometrial adenocarcinomas	None
Kataoka et al. 1997 [49]	Case report	1 primary squamous cell carcinoma of the endometrium	1 (100%) HPV-31
Lai et al. 1994 [33]	Case series	18 epithelial ovarian adenocarcinomas, of which	• 9/18 (50%) HPV-16
			• 3/18 (17%) HPV-18
		• 9 were serous;	
		• 7 were mucinous;	
		• 2 were undifferentiated adenocarcinomas.	
		18 endometrial adenocarcinomas, of which	• 8/18 (44%) HPV-16
			• 3/18 (17%) HPV-18
		• 10 were adenocarcinomas;	
		• 6 were adenosquamous carcinomas;	
		• 1 was a clear-cell carcinoma;	
		• 1 was an undifferentiated carcinoma.	
Mariño-Enríquez et al. 2008 [34]	Case series	5 primary transitional cell carcinomas of the endometrium and endometrial carcinomas with transitional cell differentiation	None
Park et al. 2004 [35]	Case series	10 endometrial adenocarcinomas	None
Plunkett et al. 2003 [19]	Case–control study	50 endometrial carcinomas, of which	1/50 (2%) HPV-16
		• 38 were endometrioid;	
		• 3 were villoglandular;	
		• 1 was serous papillary;	
		• 7 were mixed adenocarcinomas;	
		• 1 was adenosquamous.	
Yang et al. 2003 [36]	Case series	46 endometrioid endometrial carcinomas	Endometrial carcinomas:
		14 endometrioid ovarian carcinomas	7/46 (15%) HPV-16
		21 serous ovarian carcinomas	
		11 mucinous ovarian carcinomas	Ovarian carcinomas:
		6 clear-cell ovarian carcinomas	• 18/56 (32%) HPV-16
		4 undifferentiated ovarian carcinomas	• 1/56 (2%) HPV-18
Zielinski et al. 2003 [37]	Case series	20 endometrial adenocarcinomas	None
Brewster et al. 1999 [38]	Case series	58 endometrioid endometrial carcinomas	None
		4 adenosquamous endometrial carcinomas	1/4 (25%) HPV-16
		3 malignant mixed mesodermal tumors of the endometrial cavity	None
		1 squamous cell carcinoma of the endometrial cavity	1 (100%) HPV-18
Wong et al. 1993 [46]	Case series	22 endometrial adenocarcinomas	1/22 (5%) HPV-16
Runnebaum et al. 1996 [39]	Case series	7 primary fallopian tube adenocarcinomas	None
Koffa et al. 1994 [40]	Case series	14 endometrial adenocarcinomas	5/14 (36%) HPV-? (11/16/18/33)
		1 clear-cell endometrial carcinoma	None
		1 uterine leiomyosarcoma	None
		5 ovarian adenocarcinomas	None
		1 ovarian serous cystadenocarcinoma	None
		1 borderline mucinous ovarian tumor	None
		1 Krukenberg tumor	None
Runnebaum et al. 1995 [41]	Case series	20 ovarian serous cystadenocarcinomas	None
		2 ovarian endometrioid adenocarcinomas	None
		2 ovarian mucinous cystadenocarcinomas	None
		1 ovarian mixed (endometrioid and serous) cystadenocarcinoma	None
		1 heterologous malignant mixed Müllerian ovarian tumor	None
		1 undifferentiated ovarian carcinoma	None
		1 granulosa cell ovarian tumor	None
Chen et al. 1999 [42]	Case series	20 ovarian carcinomas	None
Alavi et al. 2012 [20]	Case–control study	43 ovarian serous cystadenocarcinomas	2/43 (5%) HPV-? (16/18)
		7 ovarian mucinous adenocarcinomas	1/7 (14%) HPV-? (16/18)
Ingerslev et al. 2016 [11]	Case series	146 ovarian serous adenocarcinomas	1/146 (1%) HPV-18
		10 ovarian mucinous adenocarcinomas	None
		12 ovarian endometrioid adenocarcinomas	None
		6 ovarian clear-cell carcinomas	None
Dadashi et al. 2017 [21]	Case–control study	25 ovarian carcinomas	25/70 (36%) HPV-16
Hassan et al. 2017 [43]	Case series	100 ovarian carcinomas	• 5/100 (5%) HPV-16
			• 4/100 (4%) HPV-18
			• 1/100 (1%) HPV-33
Kisseljova et al. 2020 [12]	Case series	29 ovarian serous adenocarcinomas	18/34 (53%) HPV-16
		2 ovarian mucinous adenocarcinomas	
		3 ovarian endometrioid adenocarcinomas	
Yang et al. 2020 [44]	Case series	310 ovarian carcinomas,	78/310 (25%) HPV-?
		of which	
		• 208 were serous ovarian adenocarcinomas;	
		• 48 were mucinous ovarian adenocarcinomas.	
Jarych et al. 2024 [45]	Case series	33 serous ovarian adenocarcinomas	• 14/46 (30%) HPV-16
		5 borderline ovarian tumors	• 9/46 (20%) HPV-18
		3 clear-cell ovarian adenocarcinomas	• 7/46 (15%) HPV-16+18
		3 mucinous ovarian adenocarcinomas	
		2 other types of epithelial ovarian adenocarcinomas	
Lai et al. 1992 [22]	Case–control study	11 epithelial ovarian carcinomas, of which	• 2/11 (18%) HPV-16
			• 3/11 (27%) HPV-18
		• 7 were serous ovarian carcinomas;	
		• 3 were mucinous ovarian carcinomas;	
		• 1 was a mixed ovarian carcinoma.	
		8 endometrial adenocarcinomas	2/8 (25%) HPV-16
Mohamed et al. 2024 [23]	Case–control study	47 epithelial ovarian carcinomas, of which	• 6/47 (13%) HPV-16
			• 1/47 (2%) HPV-18
		• 15 were high-grade serous carcinomas;	• 5/47 (11%) HPV-?
		• 11 were papillary serous adenocarcinomas;	
		• 10 were endometrioid adenocarcinomas;	
		• 4 were undifferentiated carcinomas;	
		• 2 were squamous cell carcinomas;	
		• 2 were mucinous borderline tumors (atypical proliferative mucinous tumors);	
		• 2 were adult-type granulosa cell tumors;	
		• 1 was a mixed malignant mullerian tumor.	

## Abbreviations

The following abbreviations are used in this manuscript:

DNA	Deoxyribonucleic Acid
EC	Endometrial Cancer
FAS	Fatty Acid Synthase
FFPE	Formalin-Fixed Paraffin-Embedded
GBM	Glioblastoma Multiforme
GLOBOCAN	Global Cancer Observatory
GST	Glutathione S-Transferase
HLA	Human Leukocyte Antigen
HPV	Human Papillomavirus
IARC	International Agency for Research on Cancer
MDM2	Mouse Double Minute 2 homolog
mRNA	Messenger Ribonucleic Acid
NSCLC	Non-Small Cell Lung Cancer
OC	Ovarian Cancer
PCR	Polymerase Chain Reaction
PFTC	Primary Fallopian Tube Carcinoma
PPC	Primary Peritoneal Carcinoma
PRISMA	Preferred Reporting Items for Systematic Reviews and Meta-Analyses
RCT	Randomized Controlled Trial
TZ	Transformation Zone
WHO	World Health Organization
